# Thermal erosion of cratonic lithosphere as a potential trigger for mass-extinction

**DOI:** 10.1038/srep23168

**Published:** 2016-03-24

**Authors:** Jean Guex, Sebastien Pilet, Othmar Müntener, Annachiara Bartolini, Jorge Spangenberg, Blair Schoene, Bryan Sell, Urs Schaltegger

**Affiliations:** 1 Institute of Earth Sciences, University of Lausanne, Géopolis, 1015 Lausanne, Switzerland; 2 Muséum National d’Histoire Naturelle, CNRS UMR 7207 Paleobiodiversité et Paléoenvironnements, CP38, 8 rue Buffon, F-75005 Paris, France; 3 Institute of Earth Surface Dynamics, University of Lausanne, Géopolis, 1015 Lausanne, Switzerland; 4 Department of Geosciences, Princeton University, 219 Guyot Hall, Princeton, New Jersey 08544, USA; 5 Earth & Environmental Sciences, University of Geneva, Rue des Maraîchers 13, 1205 Geneva, Switzerland

## Abstract

The temporal coincidence between large igneous provinces (LIPs) and mass extinctions has led many to pose a causal relationship between the two. However, there is still no consensus on a mechanistic model that explains how magmatism leads to the turnover of terrestrial and marine plants, invertebrates and vertebrates. Here we present a synthesis of ammonite biostratigraphy, isotopic data and high precision U-Pb zircon dates from the Triassic-Jurassic (T-J) and Pliensbachian-Toarcian (Pl-To) boundaries demonstrating that these biotic crises are both associated with rapid change from an initial cool period to greenhouse conditions. We explain these transitions as a result of changing gas species emitted during the progressive thermal erosion of cratonic lithosphere by plume activity or internal heating of the lithosphere. Our petrological model for LIP magmatism argues that initial gas emission was dominated by sulfur liberated from sulfide-bearing cratonic lithosphere before CO_2_ became the dominant gas. This model offers an explanation of why LIPs erupted through oceanic lithosphere are not associated with climatic and biotic crises comparable to LIPs emitted through cratonic lithosphere.

There are currently two main hypotheses to explain recurrent catastrophic global climatic change and related mass extinctions in Earth history. The first invokes super-greenhouse conditions due to extreme atmospheric CO_2_ concentrations^1^,^2^. This enrichment is often interpreted as degassing of magmatic CO_2_ from volcanic basalt provinces^3^ and/or from the degassing of carbonaceous or organic-rich sediments by sill and dyke intrusions^4^,^5^. The second scenario invokes a short period of global icehouse conditions caused by degassing of large volumes of volcanic SO_2_, atmospheric poisoning, cooling, and eustatic regression^6^,^7^. Although both hypotheses are compatible with massive volcanic degassing, they must also be able to explain the paleontological record in marine and continental stratigraphic sections. Mass extinction events are recorded in various marine and continental sedimentary sections distributed around the world. One major challenge is to correlate these sections in order to obtain a global “picture” required to understand global climate change associated to mass extinction. Marine versus continental sedimentary sections do not necessarily record the same processes neither the same time interval. In particular, LIPs volcanic activity related to major periods of extinction (Siberian Trap, CAMP, Karoo-Ferrar, Deccan) is restricted to continental settings while key observations to demonstrate global mass extinction is recorded in marine environments. It requires, therefore, precise and accurate time constraints to link volcanic activity with the change in the paleontological record, precision at the 100 ka level, which is only achievable using high precision U/Pb geochronology on zircon^8^,^9^. Here, we review data from the stratigraphic record of the Triassic-Jurassic (T-J) and Pliensbachian-Toarcian boundaries combined with geochronological data^8^,^9^,^10^ in order to establish the sequence of events that initiate two of the major mass extinctions recorded in Earth’s history. Then, the various alternatives to explain the climatic and chemical changes associated to these sequences of events are discussed.

## Sequence of events associated to the Triassic-Jurassic boundary

**Figure 1:**
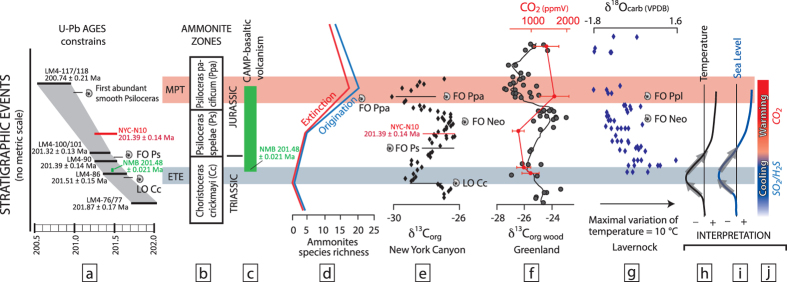
Stratigraphic and isotopic correlations for the Triassic-Jurassic boundary. (**a**) U-Pb ages on zircon from ash beds embedded in marine stratigraphic sections from N. Peru and Nevada^8^. (**b**) Standard ammonite zones for the late Triassic-early Jurassic. (**c**) Duration of CAMP volcanism in North America and Morocco (Argana Basin)^9^. (**d**) Ammonite biodiversity around the T-J boundary. (**e**) δ^13^C_org_ from New York Canyon (Nevada, USA)^11^. (**f**) δ^13^C_org_ recorded in stratigraphic sequences from East Greenland and *p*CO_2_ estimated for the late Triassic-early Jurassic^1^,^2^,^15^. (**g**) δ^18^O measured on Oysters from Lavernock (UK)^13^. (**h**) Sea level variations^8^. (**i**) Variations of the temperature^15^. (**j**) Proposed model for the decoupling between sulfur emissions related to the thermal-erosion of the continental lithosphere following by CO_2_ degassing associated to CAMP volcanism. Notes: ETE: End Triassic Extinction. MPT: Early Jurassic major plant turnover^1^. LO: Last Occurrence. FO: First Occurrence. Neo & Ppl: *Neophylites* & *Psiloceras planorbis* ammonite species respectively. (See supplementary information for additional details about the correlation between marine and continental sections).

Detailed ammonite biostratigraphy^7^,^11^ combined with the carbon and oxygen stable isotope records^1^,^12^,^13^ and zircon geochronology^8^,^9^ allows reconstruction of the sequence of events associated with biological crisis observed at the T-J boundary (Fig. 1). Figure 1 shows the synchronicity, initially pointed out by Marzoli *et al.*^14^, between end-Triassic extinction (ETE) marked by the major extinction of the Triassic ammonoids (indicated by the last occurrence (LO) of *Choristoceras crickmayi*) and the earliest CAMP volcanism^7^,^8^,^9^. The ETE event is characterized by a strong negative excursion of δ^13^C_org_ recorded in New York Canyon (Nevada, USA) and worldwide^7^,^8^ correlated to a initial negative excursion of δ^13^C_wood_ measured in fossil leaf and wood from East Greenland^1^,^12^,^15^ and by a marine regression in the upper Rhaetian of Austria, England and Nevada (Fig. 1). This eustatic regression which predates or is concomitant with environmental changes at the Rhaetian – Hettangian transition is supported by various stratigraphic evidence: (1) At New York Canyon, the end Triassic sediments are characterized by a bed packed with small shallow water bivalves indicating low sea level conditions; (2) In northern Peru (Chilingote, Utcubamba Valley) an erosional tempestite horizon located just below *Odoghertyceras* ammonite (early Jurassic) bearing sediments indicates this section was above storm wave base in the topmost Triassic^16^; (3) A pronounced increase in the proportions of terrestrial versus marine palynomorphs during the end-Triassic interval is observed in various sedimentary sequences from the Alps^17^,^18^,^19^; (4) In England, wave-ripple structures and mudcracks horizons, evidencing shallowest water depth conditions and sporadic emersions, are observed just few centimeters below the end-Triassic negative δ^13^C excursion^20^. This regressive event interpreted as cool climate conditions (Fig. 1) is in agreement with the high δ^18^O values as measured in Oysters from Lavernock (UK)^13^,^19^.

The time span between the extinction of the last Triassic ammonoids and the first occurrence of *Psiloceras spelae* Guex which defines the base of the Jurassic and the onset of recovery in marine environment, lasted for c. 90 to 200 kyr, based on sedimentary rates constrained by U-Pb zircon geochronology from ash beds in Northern Peru^8^,^11^. The ammonoid recovery during the early Jurassic is associated with progressive increase of δ^13^C_org_, decrease of δ^18^O and a significant sea level rise illustrated by the deposition of silty sediments overlying the bivalve-rich bed in the Muller Canyon Member (New York Canyon, NV). This sea level rise suggests a global warming potentially associated with large volcanic CO_2_ emissions from CAMP volcanism (Fig. 1)^1^,^2^,^12^,^15^. This warming period is associated with an abrupt change in plant diversity observed in Greenland^1^,^15^ and with a second negative δ^13^C recorded in the Hettangian *Psiloceras planorbis* beds (coeval with *P. pacificum*) that postdate the ETE (see supplementary information).

## Sequence of events associated to Pliensbachian-Toarcian boundary

**Figure 2:**
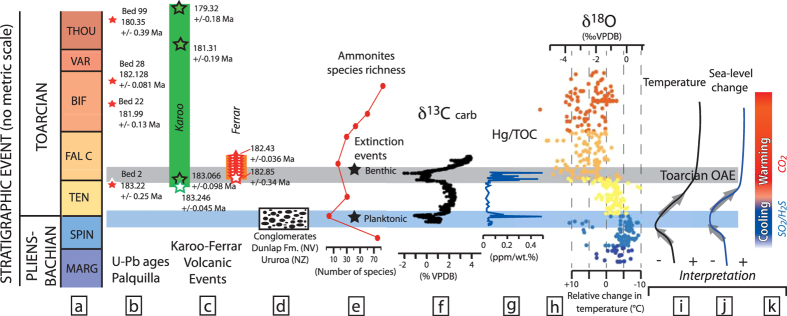
Stratigraphic and isotopic correlations for the Pliensbachian-Toarcian boundary. (**a**) Ammonite zones for the late Pliensbachian – early Toarcian. (**b**) U-Pb ages from ash beds collected at Palquilla (Peru)^10^. (**c**) U-Pb ages of the Karoo and Ferrar sills and lavas^10^,^22^. (**d**) Fluvial conglomerate deposits in Nevada and New Zealand^30^,^31^. (**e**) Variations of the biodiversity of ammonites during the Pliensbachian- Lower Toarcian^23^. (**f**) Variations of δ^13^C in the Peniche section^80^. (**g**) Variations of Hg/TOC (TOC: total organic carbon) in the Penische section^72^. (**h**) Variations in δ^18^O of belemnites (color-coded as function of their Ammonite zone location) and relative changes in temperature^27^. (**i**) Temperature variations around the Pliensbachian-Toarcian boundary. (**j**) Sea level change interpreted based on δ^18^O variation and on stratigraphic constraints. (**k**) Proposed model for the decoupling between sulfur emissions related to the thermal-erosion of the cratonic lithosphere following by CO_2_ degassing associated to Karoo-Ferrar dike swarm emplacement and volcanism. Ammonite zones: THOU: *Thouarsense*; VAR: *Varabilis*; BIF: *Bifrons*; FAL C: *Falciferum*; TEN: *Tenuicostatum*; Spin = *Spinatum*; Marg = *Margaritatus*. Notes: Toarcian OAE: Toarcian Oceanic Anoxic Event.

Environmental perturbations related to the early Jurassic Pliensbachian-Toarcian boundary have been associated for some time with the onset of the Karoo-Ferrar large igneous province^21^. This inference is confirmed by high precision U-Pb dating on zircons^10^,^22^ which indicate that major sills intruded into organic-rich sediments in the Karoo basin are correlated with the Toarcian Oceanic Anoxic Event (OAE) recorded in the marine sedimentary section from Peru, dated by ammonites and intercalated zircon bearing ash beds (Fig. 2). The end-Pliensbachian extinction, preceding the Toarcian OAE^23^, is marked by an important diversity drop (disappearance of 90% of the ammonite taxa) associated with a generalized sedimentary gap linked to a marked regression event in NW-Europe and the Pacific area^24^. This regression may represent a major short-lived glaciation associated to the growth of ice caps^25^,^26^,^27^,^28^, and was marked by important emersion topography observed on seismic images of the North Sea^24^, evidence of polar ice storage^29^ and by thick fluvial conglomerates (Dunlap Formation in Nevada (USA)^30^ and Ururoa-Kawhia area, New Zealand^31^) deposited in open marine sediments containing Late Pliensbachian ammonites (Nevada) and below sediments containing Early Toarcian ammonites (Nevada and New Zealand). The cooling model is also supported by recent δ^18^O data on belemnites^27^.

The regression phase is followed by a worldwide transgression during the Early Toarcian, with the deposition of black shales associated with the Toarcian OAE^32^. This transgression is interpreted as partly linked to glacioeustatic sea-level rise associated to rapid change from cold to warm climatic conditions^33^. The lower limit of the Toarcian OAE in S. Peru has been dated at 183.22 ± 0.25 Ma, coinciding with the oldest Karoo sill and lava currently dated (granophyre sill intruded in the Tarkastad Formation date of 183.014 ± 0.054 Ma^10^ and granophyre in the New Amalfi Sheet date of 183.246 ± 0.045 Ma^22^). The Toarcian OAE is responsible for a second extinction affecting mainly benthic foraminifera populations and brachiopods^34^,^35^,^36^ but the ammonites were only slightly affected, mostly by a moderate drop in diversity (Fig. 2).

The stratigraphic and isotopic evidence presented here indicate that initial eustatic regression events predate or are concomitant with evidence of environmental change at the Rhaetian – Hettangian and the Pliensbachian–Toarcian transitions. The major question is: what process(es) could produce these initial regressions? The short duration of these events (<1 Ma) would be difficult to explain with tectonic processes because of the long timescales associated with heat-induced changes in lithospheric buoyancy^37^. Such long-term processes also seem unable to explain the initiation of biological crises. These regressive events are difficult to reconcile either with large initial CO_2_ degassing associated with LIPs as proposed to explain the T-J and Pl-To mass extinctions (e.g. refs 1,2) or by volatile-release (CO_2_, CH_4_, Cl_2_) from deep sedimentary reservoirs during contact metamorphism associated to dykes and sills intrusion^4^,^5^ because massive CO_2_ degassing is expected to produce super greenhouse conditions. An alternative is that volcanic emissions of large igneous provinces would release a sufficiently large volume of sulfur into the atmosphere, which could generate significant cooling^6^,^38^. Measurements of sulfur content in melt inclusions or in clinopyroxene demonstrate that S contents in LIP basaltic melts could be high (>1000 ppm of S) but also vary significantly between the different provinces (e.g. refs 39, 40, 41, 42, 43, 44). The presence of recycled oceanic or continental crustal lithology in the mantle source of LIPs or crustal contamination^3^,^5^,^39^,^45^ has been proposed to explain these high S contents regarding that the melting of the depleted MORB mantle (119 ppm of S^46^) alone seems unable to explain such high values. Here we evaluate the hypothesis that initial thermal erosion of the cratonic lithosphere due to emplacement of the CAMP and Karoo-Ferrar volcanic provinces led to initial pulses of sulfur causing global cooling and eustatic regression, which was followed by warming/transgression associated with the progressive increase of CO_2_ in the atmosphere associated to LIPs emission and metamorphic reactions in sedimentary basins^4^,^5^.

## Cratonic lithosphere as a potential sulfur reservoir

**Figure 3:**
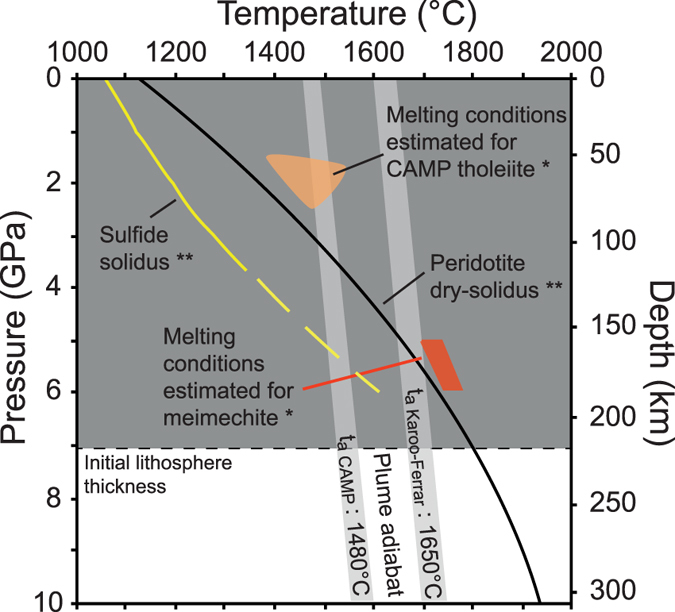
Pressure-Temperature diagrams for the condition of melting of dry-peridotite compared to the thickness of the lithosphere before thermal erosion (>200−230 km^54^,^69^). The adiabat temperatures for CAMP and Karoo-Ferrar CFB are estimated by Herzberg and Gazel^47^ based on petrological constraints. *The red and orange zones indicate the conditions of formation estimated for Vestfjalla meimechites^48^ and CAMP tholeiites^49^ respectively. **The continuous black and yellow lines show the solidus for dry peridotite^81^ and mono-sulfide solutions^70^, respectively. This latter solidus was determined only to 3.7 GPa, the yellow dashed line indicates extrapolation to higher P-T conditions (See supplementary information for details about the melting conditions in an upwelling plume).

**Figure 4:**
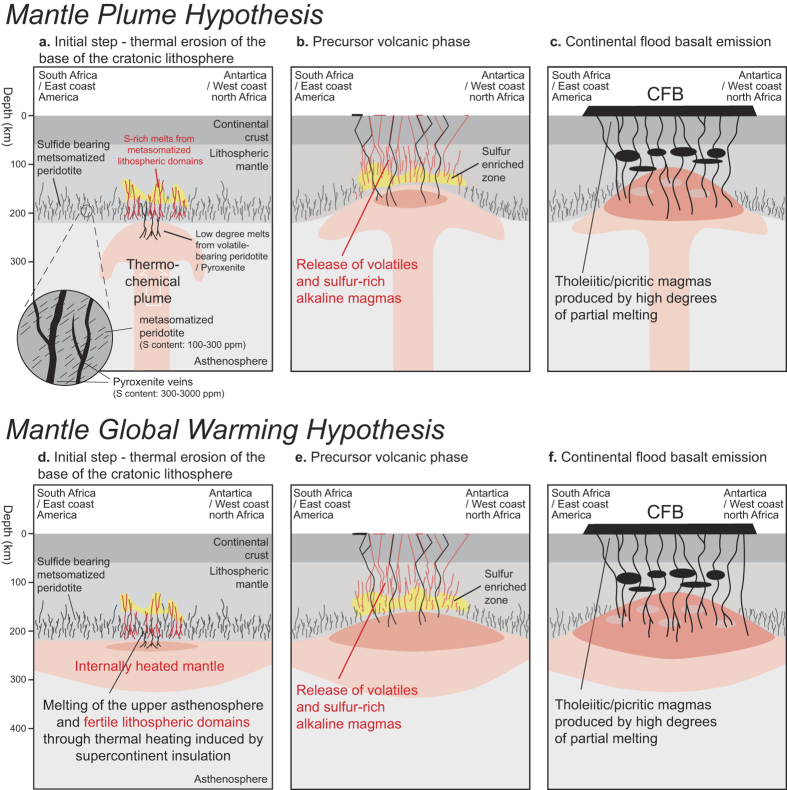
Schematic sections illustrating heating of the lithospheric mantle associated to the arrival of a thermal plume beneath a thick cratonic lithosphere (panels (**a**–**c**)) or linked to the increase of mantle temperature through supercontinent thermal insulation (panels (**d**,**e**)). The basal part of the lithosphere is composed of melt-metasomatized peridotite^69^. Panels (**a**,**d**) illustrate the initial step of melting, panels (**b**,**e**), the precursor step of magmatism suggested by Ernst and Bell^71^ while panels (**c**,**f**) show schematic sections of cratonic lithosphere during the generation of large volume of CFB. These latter sections are based on a model suggested by Heinonen *et al.*^65^ for the magma generation of Karoo-Ferrar CFB.

Petrological constraints on primary magmas indicate that the mantle is hotter and melts more extensively to produce LIP lavas or continental flood basalts (CFB) than for current oceanic islands basalts^47^. The melting of garnet-bearing peridotitic sources at high pressures (5–6 GPa) and anomalously high mantle potential temperatures (*T*_p_) of >1,600 °C (Fig. 3) are required to generate the parental melts of high-Mg# meimechites from the Karoo-Ferrar province^48^ while lower *T*_p_ (1450 °C ± 50 °C) and lower pressures of melting (>2 GPa) were estimated for tholeiitic lavas from CAMP and Ferrar large igneous province^49^. Available data suggest that the Karoo-Ferrar and CAMP have been emitted on top of thick lithosphere^50^. First, the eastern and the southern extents of the CAMP were located on top of Archean and Proterozoic cratons, while the Karoo LIP was erupted on and around the Kaapvaal craton. Shear wave velocities (V_s_), suggest that the Karoo and CAMP areas were underlain by thick lithosphere (>200 km) prior to continental break up^51^,^52^,^53^, which postdates the LIP emplacement^54^. The thin lithosphere on both margins of the CAMP area (east coast of North America and north-west coast of Africa) is the consequence of stretching and thinning before seafloor spreading^55^. A thick lithosphere exceeding 200 km beneath the Kaapvaal craton is also documented by kimberlite-borne mantle xenoliths^56^. The thickness of the lithosphere beneath the Ferrar LIP is probably similar, given that the Ferrar LIP emission was linked to the Wilkes Land craton^57^. Taking into account estimates of the thickness of continental lithosphere beneath Karoo-Ferrar and the CAMP area, the melting conditions estimated for LIP’s meimechites and tholeiitic lavas^48^,^49^ point out the fundamental role of the lithospheric mantle to produce these lavas. Various geochemical and isotopic studies on CAMP^58^,^59^,^60^ and Karoo-Ferrar magmas^61^,^62^,^63^,^64^,^65^ indicate that continental lithospheric mantle is a major geochemical component involved in the petrogenesis of these lavas. Two different hypotheses could explain the melting of the lithospheric mantle^65^: the arrival of a thermal plume initiating heating and subsequent partial melting of the subcontinental lithosphere^3^, or internal heat production of mantle insulated by continental lithosphere allowing for partial melting of the upper asthenospheric and continental lithospheric mantle to produce LIPs magmas^49^,^63^,^66^,^67^,^68^. This initial step of thermal erosion/thermal heating of the cratonic lithosphere is critical to understand the volatile budget associated with LIPs while studies of the composition of the Kaapvaal craton have shown that sulfide minerals are enclosed in the basal part of the cratonic lithosphere^69^ (Fig. 4a). The formation of these sulfide minerals are linked to multiple refertilization/metasomatic events, which affected the base of the subcontinental lithospheric mantle from the Archean to the Proterozoic^69^.

Figure 4 illustrates how thermal erosion of the lithosphere associated with the rising of a thermal plume or internal heating of the subcontinental mantle could release sulfur from the cratonic roots. (I) Low degree melts from the base of the lithosphere may rise and initiate the thermal erosion of the cratonic lithosphere (Fig. 4a,d). According to the low solidus temperature of sulfides relative to dry peridotite^70^ (Fig. 3), the sulfide-enriched lithologies will be remobilized by the progressive percolation of low degree melts from the subcontinental lithosphere. In a runaway process, the S-rich melts/fluids released from the base of the cratonic lithosphere do not directly reach the surface, but enrich progressively shallower levels of the lithospheric mantle, levels which could be remobilized sequentially during the thermal erosion of the lithosphere (Fig. 4a,d); (II) If lithospheric erosion is sufficiently shallow, scavenging of sulfur-rich melts/fluids could either reach the surface and release significant amounts of sulfur to the atmosphere or be mixed with the first CFB magma pulses producing initial high SO_2_ flux to the atmosphere (Fig. 4b,e). The former process could be linked to the emission of small volumes of alkaline lavas and carbonatites associated with translithospheric fracturing as observed in various LIP areas^71^; (III) The last stage (Fig. 4c,f) corresponds to an advanced degree of thermal erosion of the lithosphere, sufficient to lead to significant melting and extrusion of the lavas observed in the Karoo-Ferrar area and in the CAMP. The injection of large volumes of magma into sedimentary basins releases additional sediment-derived greenhouse gases (CO_2_, CH_4, …)_ produced during contact metamorphism^4^,^5^. These models are in agreement with geochemical data emphasizing an important role of the lithospheric mantle in the petrogenesis of Karoo-Ferrar and CAMP LIPs, but also suggest that a precursor magmatic phase predates the main phase of LIPs emission. The existence of a precursor phase of magmatism associated to the Karoo-Ferrar LIP is supported by recent mercury data determined in marine sediments^72^ (Fig. 2g). The high Hg concentrations and Hg/TOC (TOC: Total Organic Carbon) ratios correlated with the negative carbon-isotope anomalies associated with the Pliensbachian-Toarcian boundary and Toarcian OAE observed in Peniche, Arroyo Lapa, Mochras and Bornholm sections are indicators for two distinct episodes of Hg release to the atmosphere by volcanic activity^72^ in line with our sequence of two distinct magmatic events.

To estimate the potential amount of S released to the atmosphere during the initial phase of lithosphere thermal erosion/heating, we have calculated the amount of sulfur present in the lower 25 km of the lithosphere and assuming a surface equivalent to half the area covered by Karoo-Ferrar or CAMP LIPs. Based on garnet chemistry from Kimberlite xenoliths, the basal part of southern African lithospheric mantle includes significant proportions of melt metasomatized peridotites^56^. Assuming that the basal part of the lithosphere was composed of 40% depleted peridotite (119 ppm S), 55% metasomatized peridotite (300 ppm S) and 5% pyroxenite (800 ppm S), we obtain a potential sulfur content that could be released to the atmosphere (calculated in equivalent SO_2_) of ~45,000 Gt and ~210,000 Gt of SO_2_ for the case of the Karoo-Ferrar and CAMP, respectively (see supplementary information for details). Our estimates for potential sulfur release from the lithospheric mantle are 5 orders of magnitude higher than the mass estimated for Laki (~122 MT SO_2_^73^) or Pinatubo (~20 Mt SO_2_^74^) eruptions. Assuming that only a small proportion of this sulfur is effectively released to the atmosphere, it could still have a significant impact on climate and environment.

Thermomechanical modeling for plume-lithosphere interaction^3^ has shown that gas release from plume material could reach the surface prior to the emission of the first lavas, but this model does not take into account the gas released from the cratonic lithosphere. We suggest here that the initial gas release to the atmosphere is not solely composed by gas from the asthenosphere itself (CO_2_/HCl^3^), but additional sulfur gases produced from the thermal erosion of the cratonic lithosphere represent a significant portion of emitted gases during this initial period. Studies of recent catastrophic eruptions demonstrate that large SO_2_ emission affects the climate, but the gas flux, the latitude of the eruption and the maximum altitude reached by the volcanic clouds (troposphere or stratosphere) influences the climatic response (e.g. refs 73,75,76). Models for SO_2_ release into the atmosphere demonstrate that it is not the magnitude of SO_2_ emission, which is critical to produce cooling, but the frequency and the duration of SO_2_ emission^38^. Although we are unable to provide either a quantitative flux estimate or predictions about the heights reached by the volcanic clouds, we provide feasibility tests. Assuming that only 20% of the sulfur from the metasomatized lithospheric mantle is released to the atmosphere during a period of 100,000 years, we obtain a flux between 90 and 410 Mt/yr for the case of Karroo and CAMP, respectively. Timmreck *et al.*^77^ have modeled that sulfur emissions of ~100 times the mass of Pinatubo (corresponding to our estimate of S emitted for a period of 5 to 20 years) creates an average temperature decrease of −3.5 °K lasting for 9–10 years. Robock^75^ hypothesized that a series of volcanic eruptions could initiate a longer-term cooling period. Schmidt *et al.*^38^ support this hypothesis but indicate that SO_2_ release could cause biotic crisis only if such gas release is high and sustains for several centuries. Multiple SO_2_ pulses lasting for several thousands of years as suggested here offers a viable hypothesis for the initial cooling required to explain the eustatic regression events and provides stress conditions to initiate biological crises recorded at the end of the Rhaetian (Fig. 1) and of the Pliensbachian (Fig. 2). Nevertheless initial stress conditions do not affect all species in the same manner as illustrated in Figs 1 and 2. For example, if the Pliensbachian–Toarcian boundary is associated to ammonite and planktonic massif turn over, benthic organisms seem less affected (Fig. 2). These observations are in agreement with results from Schmidt *et al.*^38^ which indicate that the environmental effects of SO_2_ degassing could be variable depending on the ecosystem and location. Since the residence time of sulfur in the atmosphere is limited^6^,^73^,^75^,^77^, the following effects of CO_2_, CH_4_, Cl_2_ associated to flood basalt volcanism and release from contact metamorphic sediments start to overwhelm the sulfur effect and will be the dominant gas producing greenhouse conditions recorded in the paleontological records. We hypothesize that this last stage is responsible for the second extinction observed at the T-J and Pl-To boundaries. Figures 1 and 2 indicate that the Early Jurassic major plant turnover and the Toarcian OAE slightly postdate the first documented lavas or sills in CAMP and Karoo LIPs, respectively.

**Figure 5:**
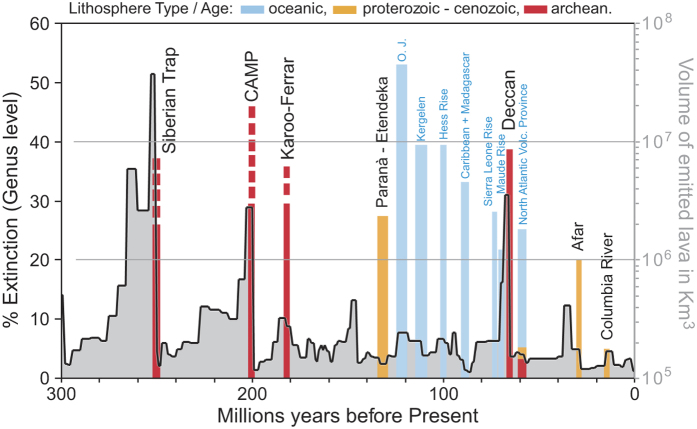
Illustration of mass extinction intensity associated with continental LIPs and oceanic plateaus against geological time. The band size related to the continental LIPs or oceanic plateaus represents the estimated volume (in km^3^) of emitted lavas^82^,^83^,^84^ plotted as a function of their respective age. The extinction intensity is defined by Rhode and Muller (ref. 85). The continental LIPs are color coded as function of the age of the underlying lithosphere (lithosphere ages from ref. 50).

The mechanisms and timescales over which sub-lithospheric mantle melts interact with thick Archean or Proterozoic continental lithosphere is therefore a major component in the timing of mass extinction processes for the case of Rhaetian/Hettangian and Pliensbachian/Toarcian boundaries. Figure 5 shows that LIPs emitted on continents have much more severe biotic crises than oceanic LIPs despite their comparable size. This figure also indicates that LIPs emitted on top of Proterozoic to Cenozoic lithosphere have limited effects relative to continental LIPs erupted on cratonic lithosphere such as the Siberian Trap, CAMP, Karoo-Ferrar and Deccan. This indicates that the nature of the underlying lithosphere during large LIP eruption potentially exerts an important control on the consequences on life at the Earth’s surface and is a viable hypothesis in addition to external causes such as asteroid impacts as proposed to explain the Cretaceous-Paleogene crisis^78^). Indeed, higher sulfur contents in CAMP and Deccan magmas, with respect to the Paraná LIP have been reported^39^. These higher magmatic sulfur contents were explained by the presence of slab-metasomatized mantle in the source of CAMP and Deccan magmas^39^. While the CAMP and Deccan LIPs were associated to major extinctions, the emission of the Paraná - Etendeka province was not^79^. Archaean lithospheric mantle is known to have suffered multiple subduction-related processes, so a difference in the nature of the underlying lithosphere (Archean in the case of CAMP and Deccan traps, Proterozoic for Paraná) provides an alternative explanation for the difference in sulfur content. The proposed mechanism also offers an explanation why large LIPs erupted over oceanic lithosphere, such as the Ontong Java or the Caribbean plateaus, did not cause climatic and biotic crises as large as those of the Karoo-Ferrar or the CAMP despite having erupted similar volumes of basaltic rocks.

## Additional Information

**How to cite this article**: Guex, J. *et al.* Thermal erosion of cratonic lithosphere as a potential trigger for mass-extinction. *Sci. Rep.* **6**, 23168; doi: 10.1038/srep23168 (2016).

## Supplementary Material

Supplementary Information
