# Supplementary material for: Thermal erosion of cratonic lithosphere as a potential trigger for mass-extinction
Source: Sci Rep. 2016 Mar 24;6:23168. doi: 10.1038/srep23168 (PMC4806358; doi:10.1038/srep23168)
Supplement: Supplementary Information [file srep23168-s1.pdf]

SCIENTIFIC REPORTS

Thermal erosion of cratonic lithosphere as a potential trigger for mass-extinction

Jean Guex[1], Sebastien Pilet[1*], Othmar Muntener[1], Annachiara Bartolini[2], Jorge Spangenberg[3], Blair Schoene[4], Bryan Sell[5], Urs Schaltegger[5]

[1] Institute of Earth Sciences, University of Lausanne, Géopolis, 1015 Lausanne, Switzerland
[2] Muséum National d'Histoire Naturelle, CNRS UMR 7207 Paleobiodiversité et Paléoenvironnements, CP38, 8 rue Buffon, F-75005 Paris, France.
[3] Institute of Earth Surface Dynamics, University of Lausanne, Géopolis, 1015 Lausanne, Switzerland
[4] Department of Geosciences, Princeton University, 219 Guyot Hall, Princeton, New Jersey 08544, USA
[5] Section of Earth & Environmental Sciences, University of Geneva, Rue des Maraîchers 13, 1205 Geneva, Switzerland

* Correspondence and requests for materials should be addressed to S.P. (email: Sebastien.Pilet@unil.ch)

SUPPLEMENTARY INFORMATION

PART I. Supplementary discussion concerning the stratigraphic correlation associated with the timing of sea level changes, $\delta^{13}C$, $\delta^{18}O$, pCO_2 variations, paleotemperatures, and the age of the onset of the CAMP-related basaltic extrusions

One of the major challenges for the worldwide stratigraphic correlation of the Triassic Jurassic (T-J) and Pliensbachian Toarcian (Pl-To) boundaries is to correlate the continental and marine sedimentary record. We present a synthetic stratigraphic correlation for marine and continental settings with the T-J correlation shown in figure 1 of the main article.

Rhaetian - Hettangian boundary

1) Correlation between marine and continental sections

Figure SI-1 illustrates the correlation between marine and continental sections of the Rhaetian-Hettangian boundary. The synthesis of the Late Rhaetian and Hettangian ammonite distribution[86] allows for a biostratigraphic correlation of marine environments. The biostratigraphically resolved carbon isotope data[87-88] can be related to the continental carbon isotope record from East-Greenland[89-90] (Fig. SI-1). U-Pb data on zircon from ash beds embedded in the Late Rhaetian - Early Hettangian of marine sequences in Northern Peru and central Nevada (USA) provide absolute time constraints for the Rhaetian-Hettangian transition that can be linked to the onset of CAMP basalt activity[86,91]. Schoene et al. (ref. 91) have demonstrated the synchroneity between the eruption of the oldest North Mountain Basalt flows, and the onset of the index fossil of the base of the Jurassic, *Psiloceras spelae* and end-Triassic biological crisis recorded in marine sediments.

An open question is whether the end-Triassic extinction (ETE) defined by Olsen et al. (ref. 92) in the continental environment represents the same event marked in the oceans by the disappearance of Triassic ammonites. Olsen et al. (ref. 92) and Blackburn et al. (ref. 93) suggested that ETE is marked by a dramatic turnover in fossil pollen, spores (sporomorphs), and vertebrates observed in early Mesozoic basins of eastern North America. However, the stratigraphy of the Rhaetian in the Newark supergroup and Argana basin is probably incomplete and contains one or several hiatuses. Kozur and Weems (refs 94, 95) were first to correlate the conchostracan biostratigraphy of the Newark Supergroup with the Germanic Triassic. They concluded that most of the Rhaetian in the Newark

Basin and elsewhere in the Newark Supergroup is missing (see also Lucas et al.; ref. 96). Gallet et al. (ref. 97) reached similar conclusions on the basis of paleomagnetic data. Therefore, the LO of *Patinasporites* interpreted as an End Rhaetian biostratigraphical marker[93] could potentially occur in the lowermost Rhaetian[98,99]. Such an interpretation is supported by the palynological study of Tethyan sections around the Norian Rhaetian boundary[99]. The palynological assemblage including *Patinasporites* is typical of the Norian- Rhaetian transition[99], and *Patinasporites* is known only at very base of the Rhaetian[100]. This suggests that the Newark fern spike is possibly lowermost Rhaetian in age rather than Rhaetian-Hettangian[99,100] (see Wotzlaw et al., ref. 101, for a discussion of the duration of the Rhaetian). In conclusion, it is not clear whether the end-Triassic biological crisis as recorded in the Newark basin by Olsen et al. (ref. 92) and Blackburn et al. (ref. 93) represents the same event recorded in other marine sequences, such as in Northern Peru and central Nevada (USA), but it does not modify the conclusion of figures 1 and SI-1, which shows that ETE is associated to a clear first negative peak in $\delta^{13}C$ and is concomitant or slightly predates the onset of CAMP.

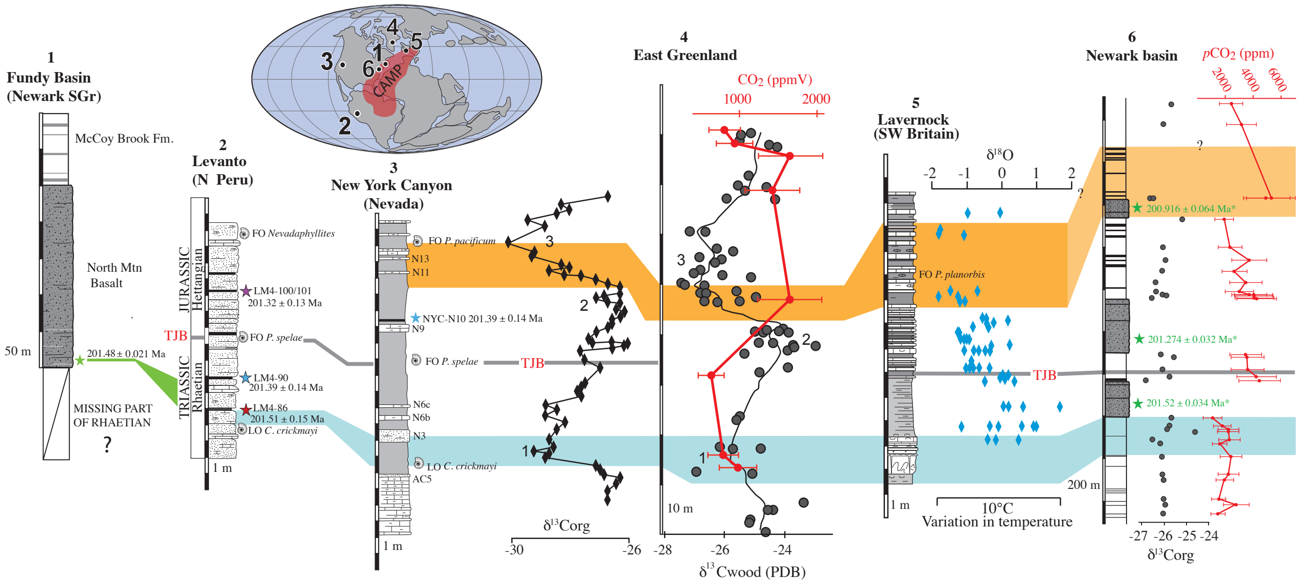

Fig. SI-1. Correlation of Rhaetian-Hettangian marine sections (Levanto, New York Canyon, Lavernock) with continental records (Newark supergroup, East Greenland). These correlations are constrained by ammonite biostratigraphy, $\delta^{13}C_{org}$, $\delta^{13}C_{wood}$ from the East Greenland continental record, and U/Pb zircon ages from Levanto and NYC ash beds (red and blue stars; ref. 91) and from zircon from Newark basin lavas (green stars, column 6: refs 91,93 (data from ref. 93 are indicated with an *). The numbers 1 to 3 highlight the proposed correlations between carbon isotope excursions [88]. Data references: (1) Fundy Basin – ref. 91; recalculated with new Earthtime spike constants reported in ref 93. (2) Levanto (N. Peru) – refs 86,91. (3) New York canyon – ref. 88. (4) East Greenland – refs 89,90. (5) Lavernock (SW England) – ref. 106. (6) Newark basin – refs 93,102.

2) Relation between extinctions and onset of the CAMP

A substantial increase of atmospheric pCO_2 interpreted as a consequence of important volcanic CO_2 emissions has been documented in East-Greenland[89,90] and in the Newark Basin[102] using stomatal analysis of fossil Ginkgoales leaves and pedogenic carbonates interbedded with volcanics of the (CAMP) respectively. The analysis of Schaller et al. (ref. 102) shows that the main pCO_2 increase appears at the end of CAMP volcanism (Fig. SI-1). Precise correlations between crucial fossil groups and the δ^{13}Corg record for Rhaetian- Hettangian sections suggests that the Major Plant Turnover defined by McElwain et al. (ref. 89) is not correlated to the d^{13}Corg negative excursion observed at the end of the Rhaetian (ref. 88), but to a second negative $\delta^{13}C$ peak recorded in the Hettangian *Psiloceras planorbis* beds (coeval with *P.pacificum* beds) occurring about 600 kyr after the main ETE. The base of that second major negative excursion correlates with the lower part of the main negative carbon excursion of Hesselbo et al. (ref. 103), attributed to the *Psiloceras planorbis* beds. The second extinction event, mainly continental, is observed in the *P. pacificum* beds[88]. Note that the palynological record in the Newark basin is characterized by abundant sporomorphs of Triassic affinity overlying the Lower Jurassic CAMP basalt[99]. Note also that all the floras recorded in the Tiefengraben Member of the Lower Jurassic (i.e. above the P.pacificum beds) in the TJB stratotype at Kuhjoch (Austria)[104] already existed in the Triassic, including C.thiergartii [105]. The low $\delta^{18}O$

values measured in Oysters coexisting with *Psiloceras planorbis* at the Lavernock section[106] is in agreement with super greenhouse conditions pointed out by the high pCO_2 inferred from East-Greenland[89,90] and in the Newark Basin[102] continental sections.

Pliensbachian – Toarcian boundary

Relations between the Pl-To and Lower Toarcian OAE extinctions with the onset of the Karoo-Ferrar LIP

There is a general agreement that the activity of the Karoo Ferrar LIP is likely related to the crises that affected the marine faunas during the Late Pliensbachian to Late Toarcian interval. The volcanic pulses were short lived and discretely distributed over time[107-109], which is consistent with the hypothesis of a volcanogenic origin for the biotic crises that are recorded by the ammonites during this period. Contact metamorphism generated by repetitive intrusions of sills within the organic rich Ecca Group in the Karoo basin (South Africa) was proposed as a mechanism to produce large volumes of methane, potentially inducing super greenhouse conditions, responsible for the Early Toarcian OAE and the negative carbon isotope excursion (CIE)[110,111]. High resolution U-Pb zircon geochronology from dolerite sills in this basin indicate a mean age of 182.7 +/- 0.4 Ma for the sill intrusions[112]. This age has recently been revised by Corfu et al. (ref. 113) to 183.05 ± 0.4 Ma, based on a recalibration of the Oslo tracer solution against the EARTHTIME synthetic 100 Ma solution. A new study combining U/Pb high precision dating on a series of Karroo sills and on zircon from ash beds from the lower Toarcian Palquilla sequence (top of the tenuicostatum zone, southern Peru) allow to correlate the onset of Karoo sill emplacement with the CIE typical for the inferred Toarcian OAE[108]. Accordingly the oldest sills dated in the Karoo basin are 183.014 ± 0.054/0.072/0.21 Ma (X/Y/Z notation after Schoene et al.; ref. 114) while the age of ash bed 2 associated at the onset of the CIE for the Toarcian OAE in the Palquilla sequence is 183.22 ± 0.25/0.26/0.32 Ma [108](Fig. 2). Burgess and co-authors (ref. 109) confirm the age estimate for the early volcanism in Karoo even the obtained age on a granophyre from the New Amalfi Sheet in southeastern South Africa is slightly older that the sills dated by Sell et al. (ref. 108) – New Amalfi sheet I-247: 182.246 ± 0.045/0.066/0.21 Ma [109]. The finding of the negative CIE in the Pacific area confirms its global significance[115-117].

The end-Pliensbachian extinction, which predates the Toarcian OAE by probably several hundred kyr is marked by an important diversity drop associated with a generalized sedimentary hiatus linked to a marked marine regression in NW-Europe and the Pacific area[118]. As indicated in the main text, this regression may represent a major short-lived glaciation[119-124]. This major regression is marked by important emersion topography documented by the deposition of thick shallow marine conglomerates in the Dunlap Formation in Nevada (USA) containing the lower Toarcian ammonite *Tiltoniceras* (= Muller and Ferguson's (ref. 125) "*Harpoceras*"), and in the top Pliensbachian of the Ururoa-Kawhia area, New Zealand, just below beds with ammonites that we identified as the coeval *Dactylioceras* aff. *semicelatum* (Lower Toarcian in age)[126]. The regression related to cooling is supported by several recent $\delta^{18}O$ data on belemnites[120-122, 127].

PART II. Derivation of a model for sulfur degassing by thermal erosion of the cratonic lithosphere

To estimate the amount of SO_2/H_2S that could be released by thermal erosion of the cratonic lithosphere, we first established a mass balance for the amount of S stored in the cratonic lithosphere beneath Karoo-Ferrar and the CAMP areas. For that, we need (a) the volume of thermally erodable cratonic lithosphere and (b) the S budget of the metasomatized cratonic lithosphere.

Volume of lithosphere thermally eroded

The total surface area covered by Karoo-Ferrar and CAMP lava flows or Dike swarms are estimated to be ~2.15 x 10^6 km^2 (ref. 128 and references therein) and between ~1 x 10^7 and 11 x 10^7 km^2 (refs 129,130), respectively. In our model, we used the lower band of CAMP surface estimates.
The thickness of the Karoo-Ferrar and CAMP lithosphere is an important parameter of our interpretation. As indicated in the article, different data suggest that the Karoo-Ferrar and CAMP have been emitted on the top of an initial thick lithosphere. Figure 3 of the article compares the position of the peridotite solidus[131] with the thickness

of the lithosphere as a function of pressure and temperature. Mantle melting is controlled by the rock types present in the upwelling plume (peridotite/ pyroxenite) and their respective volatile contents. Melting of such heterogeneous mantle in an upwelling plume will start at a depth with the melting of volatile-rich peridotite followed by pyroxenites (e.g. refs. 132, 133). It is important to note, however, that the chemical composition of the initial low-degree melts produced from pyroxenitic or volatiles-rich lithologies differ significantly from LIP lavas [134,135]. The enclosing peridotite will start to melt only at relatively shallow depth. Most models for the formation of LIP lavas suggest a high degree of partial melting (e.g. ref. 136) in order to explain the formation of high-Mg basalts such as picrites or komatiites observed in some LIPs. For example, Heinonen and Luttinen (ref. 137) suggested that Vestfjella meimechites, observed in Antarctica, are produced at pressure between 5 and 6 GPa at anomalously high mantle potential temperatures (T_p>1,600°C). Hole (ref 138), using modeled primary magma composition, has estimated lower pressure (> 2 GPa) and lower Tp (1450°C ± 50°C) for CAMP and Ferrar LIPs tholeiitic lavas. For the degree of partial melting relevant to produce magma with MgO content higher that 15 wt.% (F >5%), the effect of volatiles on melting temperature is insignificant as indicated by various parameterizations (e.g. refs 139, 140). The solidus temperature for dry peridotite could therefore be used as a minimum estimate for the conditions of magma generation of continental flood basalt, independent of the mechanism of magma generation in a mantle plume[137], by thermal heating of the lithosphere[141,142] or by delamination of the lithosphere[143]. Assuming a potential adiabat temperature of 1650°C, the beginning of melting appears at a depth of ~ 175 km and temperature around ~1700°C. Taking into account estimates of the thickness of subcontinental lithosphere beneath Karoo and the CAMP area (230 km for Karoo-Ferrar based on xenoliths carried by Limpopo Kimberlite[144]) and more than 200 km for the CAMP area[145], this suggests that at least the first 25 km of subcontinental lithosphere needs to be thermally eroded to allow the mantle to produce basaltic lavas.

Estimates of potential S stored in the basal part of the lithosphere.

The table SI-1 lists the different values used to calculate the potential S stored in the basal part of the lithosphere, Sulfur that could be potentially release to the atmosphere by thermal erosion / thermal heating of the lithosphere. Figure SI-2 reports the schematic composition of the lithosphere for the Karoo area proposed by Griffin et al. (ref. 144). The metasomatic lithologies (pyroxenite veins and melt-metasomatized peridotite) are interpreted as the result of the percolation of asthenospheric melts and fluids associated with Archean and Proterozoic subduction events and intraplate magmatism[146]. One key aspect is that sulfide minerals are precipitated during these metasomatic event[146,147] and represent a S-reservoir potentially important for the formation of LIPs.

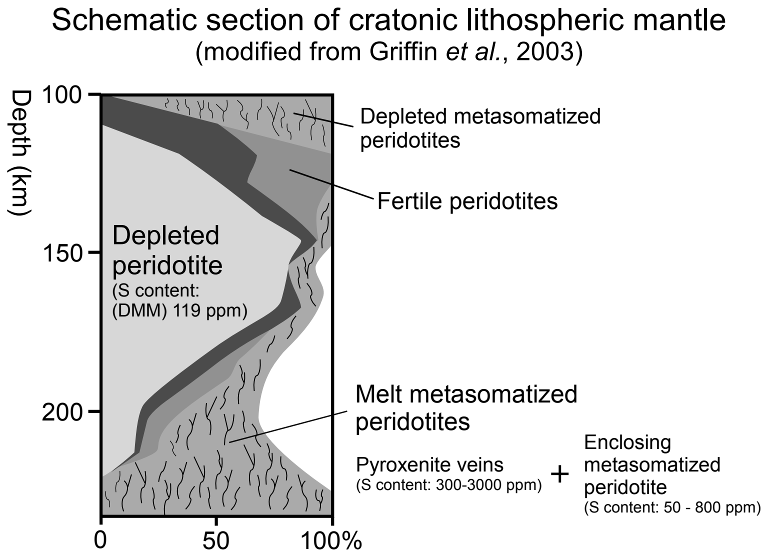

Fig. SI- 2. Subcontinental lithosphere section for southern Africa, showing the relative abundances of different peridotite types based on the study of garnet compositions from mantle xenoliths carried by kimberlites[144]. This section is based on Limpopo kimberlites erupted 500 Ma ago, i.e. before the Karoo-Ferrar volcanic activity.

For our model, we assume that the basal part of the lithosphere is composed of 45% depleted peridotite, 55% melt-metasomatised peridotite and 5% of metasomatic veins, which is known to be associated with the metasomatic enrichment of the lithosphere[148].

Griffin *et al.* (ref. 146) indicate that the Sulfur content of peridotite xenoliths is between 50 and ≥6000 ppm. If the extremely high S value observed in one xenolith seems related to late alteration, the xenoliths with equilibration temperature > 1100°C, relevant for the basal part of the lithosphere, have >300 ppm S. This value, which is in agreement with that observed in metasomatized peridotites studied by Jégo and Dasgupta (ref. 149) or measured in subcontinental peridotitic massifs (Fig. SI-3), is used in our calculation as the S content of metasomatized peridotite. A value of 119 ppm is used for the sulfur content of depleted peridotite according the S-estimate for DMM by Salters and Stracke (ref. 150). The sulfur content (800 ppm) of metasomatic veins, mostly pyroxenites, is derived from our own compilation of pyroxenite veins in orogenic peridotite massifs (Fig. SI-3), corresponding to lithosphere metasomatized by subduction zone fluids or melts.

The S content was calculated by using the concentration of S in the lithospheric mantle and the volume of the thermally eroded lithosphere. Bockrath et al. (ref. 151) have experimentally determined the sulfide solidus and liquidus in peridotite for pressures ranging between 0 and 3.3 GPa. Neglecting the fact that the P-T range was lower than the range expected for thermal heating of the lithosphere, the study indicates that the solidus temperature of $(Fe,Ni,Cu)_{1-x}$ monosulfide is lower than the solidus for anhydrous peridotite. Based on these experiments, Hart and Gaetani (ref. 152) note that '*for a reasonable mantle potential temperature (1500°C), mantle upwelling along an adiabat will intersect the sulfide solidus at ~160 km depth, and the sulfide will be fully molten before the upwelling even reaches the peridotite solidus at ~110 km*'. This suggests that, during thermal erosion or thermal heating of lithosphere, sulfides are likely to melt. Nevertheless, a S bearing phase seems to still be present even if the degree of melting reached by the peridotite is significant[153], questioning the amount of S release to the melt. Though solubility of S in silicate melts is well constrained at low pressure (e.g. ref. 154), less data are available at higher pressure. Based on a parameterization of Mavrogenes & O'Neill (ref. 155) the S solubility in basaltic melts at a depth of 110 km was estimated to be around 1000 ppm[152]. The potential transport of S during thermal erosion of the lithosphere will be significantly enhanced if fluids or sulfide liquids are produced[156]. Recent high-pressure experiments on sulfide-bearing ocean-crust indicate that, if fluids are produced at depth, such fluids could dissolve several wt. % S largely independent on fO_2 [149].

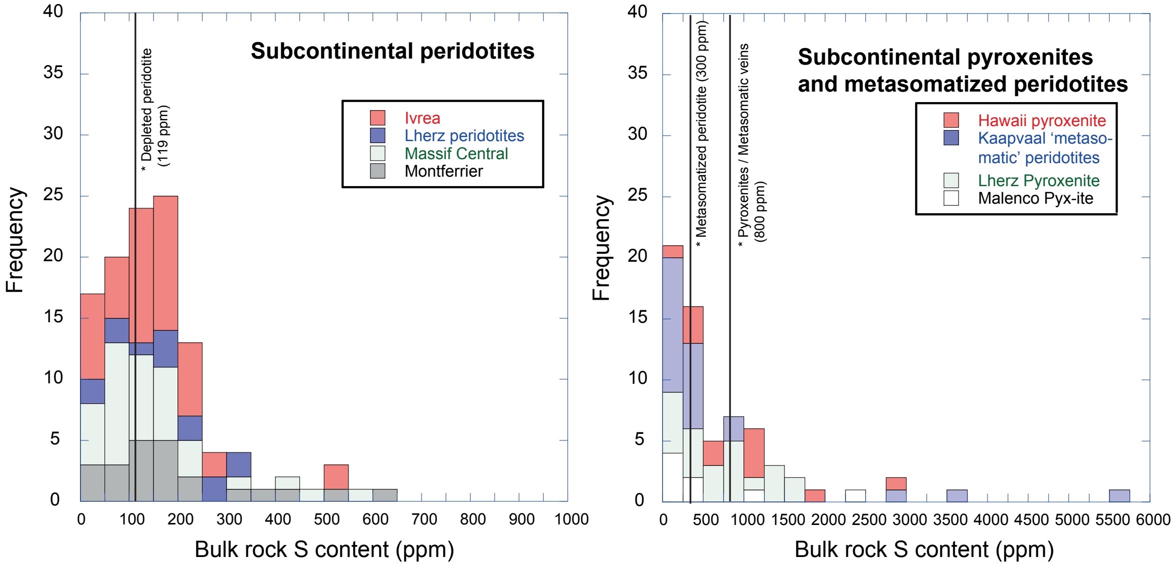

Fig. SI-3. Compilation of Sulfur contents in metasomatized peridotites[146] compared to peridotites and pyroxenites from subcontinental peridotite massifs. References for peridotite S content are: Ivrea zone – ref. 167; Lherz – ref. 168; Massif Central – refs 169,170; Peridotites Montferrier (southern France) – ref. 171. References for pyroxenites S content: Lherz – ref. 172; Hawaii – ref.; Malenco –ref. 174. * Selected values for peridotite (depleted MORB mantle[150]), metasomatized peridotite and pyroxenite.

We suggest the following dynamic model for the release of S from the metasomatized lithosphere. This model illustrated in figure 4 (main article), suggests that thermal erosion of the lithosphere is linked to the arrival of a thermal plume or by internal heating of the upper mantle[141,142]. Note that our hypothesis is still valid if other mechanisms for CFB magma production are considered such as delamination of the base of the lithosphere[143]; the thermal heating of the base of the lithosphere is required in all models to account for the formation conditions of CFB. Our model is based on thermal erosion processes such as those described in the Ronda peridotite massif (Spain) by Van der Wal & Bodinier (ref. 157); Lenoir *et al.*, (ref. 158) and Bodinier *et al.* (ref. 159), extrapolated to higher pressure and distinct (metasomatized) lithospheric mantle composition. During the thermal heating of the base of the lithosphere, initial melt is produced by melting of phlogopite-bearing lithologies and pyroxenites present in the lower part of this lithosphere, lithologies characterized by solidus temperatures significantly lower than those of anhydrous peridotite. The migration of these volatile rich melts will produce km-scale pervasive melt percolation associated with an important accumulation of melt at the recrystallisation front[157]. After several km of thermal erosion of the lithosphere, the melt present at the recrystallization front is expected to be highly enriched in volatiles including sulfur. We assume that fluids and/or sulfide liquids form at this stage. Such fluids or sulfide melts are expected to move to the surface and provide a way to emit significant S in the form of SO_2 or H_2S to the atmosphere, slightly before or concomitant with the first magma pulses produced from the asthenosphere. Jégo & Dasgupsta (ref. 149) indicate that at high pressure and for reducing conditions, the S is dissolved in fluids as H_2S. Nevertheless, during the transport and release to the atmosphere, a significant portion of the H_2S will be converted to SO_2, in particular because the oxidation state of the fluids is expected to change with decreasing pressure. (*note: we do not calculate the H_2S/SO_2 ratio of the gas flux to the atmosphere, we report the total amount of S release as SO_2* in our calculation).

Table SI-1. Estimation of the amount of S stored in the basal part of cratonic lithosphere

**Volume of lithosphere potentially thermally eroded**			
		Karoo-Ferrar	CAMP
Surface area covers by continental flood basalt	Km^2	2150000	10000000
Surface area of lithosphere potentially thermal eroded (=0.5 x Surface covers by CFB)	Km^2	1075000	5000000
Thickness of the lithospheric mantle potentially thermal eroded	Km	25	25
Volume of the lithospheric mantle potentially thermally eroded	Km^3	26875000	125000000

S content in the basal part of the lithosphere:

	Proportion of the differnt lithologies	S content (ppm)
Depleted peridotite	0.4	119 ppm
Metasomatized peridotie	0.55	300 ppm
Pyroxenite	0.05	800 ppm
**S content:**		252.6 ppm
	Convert in SO_2	505 ppm

Mass of lithospheric mantle potentially thermally eroded
(= volume of lith. x mass of 1 km^3 of lithospheric mantle (3.35×10^{12} kg)

Karoo-Ferrar	CAMP
9.003×10^{19} kg	4.188×10^{20} kg
9.003×10^{16} t	4.188×10^{17} t

Mass of SO_2 potentially releasable to the atmosphere
(= mass of lithosphere thermally eroded x SO_2 content in the lithosphere)

Karoo-Ferrar	CAMP
4.547×10^{13} t SO_2	2.115×10^{14} t SO_2
**≈ 45000 Gt SO_2**	**≈ 210000 Gt SO_2**

For comparison, the eruption of Pinatubo have release 20 Mt of SO_2 in the atmosphere (Bluth et al., ref. 161) while the Laki release 122 Mt of SO_2 (Thordarson & Self, ref. 160)

If we compare the amount of SO_2 calculated here with the estimated values for the Laki (122 MT SO_2; ref. 160) or Pinatubo (20 Mt SO_2; ref. 161) eruptions, the volumes of SO_2 potentially realizable to the atmosphere during the initial stage of Karoo or CAMP magmatism are large. As indicated in the article, we assume that only a fraction of the Sulfur initially stored in the basal part of the lithosphere is release to the atmosphere, but this could create multiples injections of SO_2 at high flux in the atmosphere during a relatively short period of time. If the timing is difficult to constrain, we assume that SO_2 release starts 0.2 to 0.4 Ma prior to the emission of CFB until massif magma eruptions. This duration is based on the thermomechanical model for plume lithosphere interaction developed for the case of Siberian Traps[162], model that indicates a time decoupling of >0.4 Ma between the first release of gases from the plume (mostly CO_2 and HCl) and the first lavas eruption. According the different studies on the climatic effect of volcanic SO_2 degasing [160,161,163-166], we hypothesize that these multiple pulses could initiate cooling of the atmosphere and global biologic crises recorded at Rhaetian - Hettangian and Pliensbachian – Toarcian boundaries.

References

86. Guex J. *et al.* Geochronological constraints on post-extinction recovery of the ammonoids and carbon cycle perturbations during the Early Jurassic. *Palaeogeography, Palaeoclimatology, Palaeoecology* **346–347**, 1-11 (2012).
87. Guex, J., Bartolini, A., Atudorei, V., & Taylor, D. High-resolution ammonite and carbon isotope stratigraphy across the Triassic–Jurassic boundary at New York Canyon (Nevada). *Earth and Planetary Science Letters* **225**, 29–41 (2004).
88. Bartolini, A. *et al.* Disentangling the Hettangian carbon isotope record: Implications for the aftermath of the end-Triassic mass extinction. *Geochemistry Geophysics Geosystems* **13 (1)**. doi:10.1029/2011GC003807 (2012).
89. McElwain, J. C., Beerling, D. J., & Woodward, F.I. Fossil plants and global warming at the Triassic-Jurassic Boundary, *Science* **285**, 1386–1390 (1999).
90. McElwain, J. C., Wagner, P.J., & Hesselbo S.P. Fossil plant relative abundances indicate sudden loss of Late Triassic biodiversity in East Greenland, *Science* **324**, 1554–1556 (2009).
91. Schoene, B., Guex, J., Bartolini, A., Schaltegger, U., & Blackburn, T. J. Correlating the end-Triassic mass extinction and flood basalt volcanism at the 100 ka level. *Geology* **38**, 387–390 (2010).
92. Olsen P. E. *et al.* Continental Triassic-Jurassic boundary in central Pangea: Recent progress and discussion of an Ir anomaly. In Koeberl, C. & MacLeod, K. G. (eds) Catastrophic Events and Mass Extinctions: Impacts and Beyond, *Geological Society of America Special Paper* **356**, 505–522. (2002).
93. Blackburn T.J. *et al.* Zircon U-Pb Geochronology Links the End-Triassic Extinction with the Central Atlantic Magmatic Province. *Science* **340**, 941-945 (2013).
94. Kozur, H., & Weems, R.E. Conchostracan evidence for a late Rhaetian to early Hettangian age for the CAMP volcanic event in the Newark Supergroup, and a Sevatian (late Norian) age for the immediately underlying beds. *Hallesches Jahrbuch Geowissenschaft* **B27**, 21–51 (2005).
95. Kozur, H., & Weems, R.E. Upper Triassic conchostracan biostratigraphy of the continental rift basins of eastern North America: its importance for correlating Newark Supergroup events with the Germanic basin and the international geologic timescale. *New Mexico Museum of Natural History and Science Bulletin* **41**, 137–188 (2007).
96. Lucas S.G., Tanner, L.H., Kozur, H.W., Weems, R.E. & Heckert, A.B. The Late Triassic timescale: Age and correlation of the Carnian–Norian boundary. *Earth-Science Reviews* **114**, 1–18 (2012).
97. Gallet, Y., Krystyn, L., Marcoux, J., & Besse, J. New constraints on the End-Triassic (Upper Norian-Rhaetian) magnetostratigraphy. *Earth and Planetary Science Letters* **255**, 458–470 (2007).
98. Cirilli, S., Upper Triassic-lowermost Jurassic palynology and palynostratigraphy: a review. In: Lucas, S.G. (Ed.), The Triassic Timescale: Geological Society, London, Special Publications, **334**, 221–262 (2010).
99. Cirilli, S. *et al.* Latest Triassic onset of the Central Atlantic Magmatic Province (CAMP) volcanism in the Fundy Basin (Nova Scotia): New stratigraphic constraints. *Earth and Planetary Science Letters* **286**, 514–525 (2009).
100. Kürschner, W.M., & Herngreen, W. Triassic palynology of central and northwestern Europe: a review of palynofloral diversity patterns and biostratigraphic subdivisions. *In:* Lucas, S.G. (Ed.), The Triassic Timescale: Geological Society, London, Special Publications, **334**, 263–283 (2010).
101. Wotzlaw J.-F. *et al.* Towards absolute time 1 calibration of the Upper Triassic: The duration of the Rhaetian. *Geology* **42**, 571-574 (2014).
102. Schaller, M.F., Wright, J.D., & Kent, D.V., Atmospheric $_{P}CO_2$ perturbations associated with the Central Atlantic Magmatic Province, *Science* **331**, 1404-1409 (2011).
103. Hesselbo S.P., Robinson, S.A., Surlyk, F., & Piasecki, S. Terrestrial and marine extinction at the Triassic–Jurassic boundary synchronized with major carbon-cycle perturbation: a link to initiation of massive volcanism? *Geology* **30**, 251–254 (2002).
104. Bonis, N. R., Ruhl, M., & Kürschner, W. M. Climate change driven black shale deposition during the end-Triassic in the western Tethys. *Palaeogeography, Palaeoclimatology, Palaeoecology* **290**, 151–159 (2009).
105. Lindström S. *et al.* No causal link between terrestrial ecosystem change and methane release during the end-Triassic mass extinction. *Geology* **40**, 531-534 (2012).
106. Korte, C., Hesselbo, S.P., Jenkyns, H.C., Rickaby, R.E.M., & Spotl, C. Palaeoenvironmental significance of carbon- and oxygen-isotope stratigraphy of marine Triassic–Jurassic boundary sections in SW Britain, *Journal of the Geological Society* **166**, 431–445 (2009).

107. Jourdan, F., Féraud, G., Bertrand, H., Watkeys, M. K., & Renne, P. R. The $^{40}Ar/^{39}Ar$ ages of the sill complex of the Karoo large igneous province: Implications for the Pliensbachian-Toarcian climate change, *Geochemistry Geophysics Geosystems* **9,** doi: 10.1029/2008GC001994 (2008).
108. Sell B., Ovtcharova M., Guex J., Jourdan F. & Schaltegger U. Evaluating the link between the Karoo LIP and climatic-biologic events of the Toarcian Stage with high-precision U-Pb geochronology. *Earth and Planetary Science Letters* **408**, 48-56 (2014).
109. Burgess, S. D., Bowring, S. A., Fleming, T. H. & Elliot, D. H. High-precision geochronology links the Ferrar large igneous province with early-Jurassic ocean anoxia and biotic crisis. *Earth and Planetary Science Letters* **415**, 90-99 (2015).
110. Polteau, S., Corfu, F., Svensen, H. & Planke, S. Rapid emplacement of the Karoo Basin sill complex during the Toarcian revealed by U-Pb dating of zircons, *in* EGU General Assembly 2010, *Geophysical Research Abstracts* **12**, (2010)
111. Aarnes, I., Svensen, H., Polteau, S. & Planke, S. Contact metamorphic devolatilization of shales in the Karoo Basin, South Africa, and the effects of multiple sill intrusions. *Chemical Geology* **281**, 181–194 (2011).
112. Svensen, H., Corfu, F., Polteau, S., Hammer, Ø. & Planke, S. Rapid magma emplacement in the Karoo Large Igneous Province. *Earth and Planetary Science Letters* **325-326**, 1–9 (2012).
113. Corfu, F., Svensen, H. & Mazzini, A. Comment to paper: Evaluating the temporal link between the Karoo LIP and climatic–biologic events of the Toarcian Stage with high-precision U–Pb geochronology by B. Sell, *et al.* in *Earth and Planetary Science Letters* 408 (2014) 48–56. *Earth and Planetary Science Letters* **434**, 349–352. (2016)
114. Schoene, B., Crowley, J. L., Condon, D. J., Schmitz, M. D. & Bowring, S. A. Reassessing the uranium decay constants for geochronology using ID-TIMS U-Pb data. *Geochimica et Cosmochimica Acta* **70**, 426-445 (2006).
115. Caruthers, A.H., Gröcke, D.R. & Smith, P.L. The significance of an Early Jurassic (Toarcian) carbon-isotope excursion in Haida Gwaii (Queen Charlotte Islands), British Columbia, Canada. *Earth and Planetary Science Letters* **307**, 19–26 (2011).
116. Gröcke, D. R, Hori, R. S., Trabucho-Alexandre, J., Kemp, D. B. & Schwark, L. An open marine record of the Toarcian oceanic anoxic event. *Solid Earth Discussion* **3**, 385–410 (2011).
117. Guex J., Bartolini A., Spangenberg J., Vicente J.-C & Schaltegger U. Ammonoid multi-extinction crises during the Late Pliensbachian – Toarcian and carbon cycle instabilities, *solid earth discussion* **4**, 1205-1228 (2012).
118. Marjanac T. & Steel, R.J. Dunlin Group sequence stratigraphy in the northern North Sea: A model for Cook sandstone deposition, *A.A.P.G. Bulletin* **81**, 276-292 (1997).
119. Guex, J., Morard A., Bartolini A. & Morettini E. Découverte d'une importante lacune stratigraphique à la limite Domérien-Toarcien : implications paléo-océanographiques, *Bulletin de la Societé Vaudoise des Sciences Naturel* **87**, 277-284 (2001).
120. Dera, G. *et al.* Watermass exchange and variations in seawater temperature in the NW Tethys during the Early Jurassic: evidence from neodymium and oxygen isotopes of fish teeth and belemnites. *Earth and Planetary Science Letters* **286**, 198–207 (2009).
121. Dera, G. *et al.* High-resolution dynamics of Early Jurassic marine extinctions: the case of Pliensbachian–Toarcian ammonites (Cephalopoda). *Journal of the Geological Society* **167**, 21-33 (2010).
122. Dera, G., Neige, P., Dommergues, J.-L. & Brayard, A. Ammonite paleobiogeography during the Pliensbachian–Toarcian crisis (Early Jurassic) reflecting paleoclimate, eustasy, and extinctions. *Global and Planetary Change* **78**, 92–105 (2011).
123. Suan, G. *et al.* Secular environmental precursors to Early Toarcian (Jurassic) extreme climate changes, *Earth and Planetary Science Letters* **290**, 448-458 (2010).
124. Suan, G. *et al.* Polar record of Early Jurassic massive carbon injection. *Earth and Planetary Science Letters* **312**, 102–113 (2011).
125. Muller, S.W. & Ferguson, H.G. Mesozoic stratigraphy of the Hawthorne and Tonopah quandrangles, Nevada. *Geological Society of America Bulletin* **50**, 1573-1624 (1939).
126. Hudson, N. Stratigraphy and correlation of the Ururoan and Temaikan Stage (Lower-Middle Jurassic, ? Sinemurian-Callovian) sequences, New Zealand, *Journal of the Royal Society of New Zealand* **33**, 109-147 (2003).
127. Gómez, J.J., Goy, A. & Canales, M.L. Seawater temperature and carbon isotope variations in belemnites linked to mass extinction during the Toarcian (Early Jurassic) in Central and Northern Spain. Comparison with other European sections. *Palaeogeography, Palaeoclimatology, Palaeoecology* **258**, 28-58 (2008).
128. Ross, P.S. *et al.* Mafic volcaniclastic deposits in flood basalt provinces: A review; *Journal of Volcanology and Geothermal Research* **145,** 281–314 (2005).
129. Knight, K.B. *et al.* The Central Atlantic Magmatic Province at the Triassic–Jurassic boundary: paleomagnetic and 40Ar/39Ar evidence from Morocco for brief, episodic volcanism. *Earth and Planetary Science Letters* **228**, 143–160 (2004).
130. McHone, J.G., Anderson, D.L., Beutel, E.K. & Fialko, Y.A., Giant dikes, rifts, flood basalts, and plate tectonics; A contention of mantle models, in Foulger, G.R., Natlund, J.H., Presnall, D.C., and Anderson, D.L., eds., Plates, Plumes, and Paradigms: *Geological Society of America*, *Special paper* **388**, 401-420 (2005).
131. Hirschmann, M.M., Mantle solidus : Experimental constraints and the effects of peridotite composition. *Geochemistry Geophysics Geosystems* **1**. doi: 10.1029/2000GC000070 (2000).
132. Hirschmann, M.M. & Stolper, E.M., A possible role for garnet pyroxenite in the origin of the 'garnet signature' in MORB. *Contributions to Mineralogy and Petrology* **124**, 185-208 (1996).
133. Ito, G. & Mahoney, J.J. Flow and melting of a heterogeneous mantle: 1. Method and importance to the geochemistry of ocean island and mid-ocean ridge basalts. *Earth and Planetary Sciences Letters* **230**, 29-46 (2005).
134. Kogiso, T., Hirschmann, M.M. & Frost, D.J. High-pressure partial melting of garnet pyroxenite: possible mafic lithologies in the source of ocean island basalts. *Earth and Planetary Science Letters* **216**, 603-617 (2003).
135. Dasgupta, R., Hirschmann, M.M. & Smith, N.D. Partial melting experiments of peridotite + CO2 at 3GPa and genesis of alkalic ocean island basalts. *Journal of Petrology* **48**, 2093-2124 (2007).
136. Herzberg, C. & Gazel, E. Petrological evidence for secular cooling in mantle plumes. Nature **458**, 619-622 (2009).
137. Heinonen, J. S. & Luttinen, A. V., Mineral chemical evidence for extremely magnesian subalkaline melts from the Antarctic extension of the Karoo large igneous province. *Mineralogy and Petrology* **99**, 201–217 (2010).
138. Hole, M. J. The generation of continental flood basalts by decompression melting of internally heated mantle. *Geology* **43**, 311-314 (2015).
139. Katz, R.F., Spiegelman, M. & Langmuir, C.H., A new parameterization of hydrous mantle melting. *Geochemistry, Geophysics, Geosystems* **4**, 1073, doi: 10.1029/2002GC000433 (2003).
140. Dasgupta, R., Hirschmann, M. M. & Smith, N. D. Water follows carbon: CO2 incites deep silicate melting and dehydration beneath mid-ocean ridges. *Geology* **35**, 135-138 (2007).

141. Coltice N., Phillips B.R., Bertrand H., Ricard Y. & Rey P. Global warming of the mantle at the origin of flood basalts over supercontinents. *Geology* **35**, 391–394 (2007).
142. Coltice, N. *et al.* Global warming of the mantle beneath continents back to the Archaean. *Gondwana Research* **15**, 254-266 (2009).
143. Elkins-Tanton L.T. Continental magmatism caused by lithospheric delamination. In: Foulger GR, Natland JH, Presnall DC, Anderson DL (eds) Plates, plumes and paradigms. *Geological Society of America*, *Special paper* **388**, 449–462 (2005).
144. Griffin, W.L., O'Reilly, S.Y., Natapov, L.M. & Ryan, C.G. The evolution of lithospheric mantle beneath the Kalahari Craton and its margins. *Lithos* **71**, 215–241 (2003).
145. McKenzie, D. & Priestley, K. The influence of lithospheric thickness variations on continental evolution. *Lithos* **102**, 1–11 (2008).
146. Griffin, W.L., Graham, S., O'Reilly, S.Y. & Pearson, N.J. Lithosphere evolution beneath the Kaapvaal Craton: Re–Os systematics of sulfides in mantle-derived peridotites. *Chemical Geology* **208**, 89–118 (2004).
147. O'Reilly, S. Y. & Griffin, W. L. Imaging global chemical and thermal heterogeneity in the subcontinental lithospheric mantle with garnets and xenoliths: Geophysical implications. *Tectonophysics* **416**, 289-309 (2006).
148. Harte, B., Hunter, R. H. & Kinny, P.D. Melt geometry, movement and crystallization, in relation to mantle dykes, veins and metasomatism. *Philosophical Transaction of the Royal Society of London,* Series A **342**, 1-21 (1993).
149. Jégo, S. & Dasgupta, R. Fluid-present melting of sulfide-bearing ocean-crust: Experimental constraints on the transport of sulfur from subducting slab to mantle wedge. *Geochimica et Cosmochimica Acta* **110**, 106–134 (2013).
150. Salters, V.J.M. & Stracke, A. Composition of the depleted mantle. *Geochemistry, Geophysics, Geosystems* **5**, Q05004, doi:10.1029/2003GC000597 (2004).
151. Bockrath C., Ballhaus C. & Holzheid A. Fractionation of the platinum-group elements during mantle melting. *Science* **305**, 1951–1953 (2004).
152. Hart, S.R. & Gaetani, G.A. Mantle Pb paradoxes: the sulfide solution, *Contributions to Mineralogy and Petrology* **152**, 295–308 (2006).
153. Luguet, A., Shirey, S.B., Lorand, J.-P., Horan, M.F. & Carlson, R.W. Residual platinum-group minerals from highly depleted harzburgites of the Lherz massif (France) and their role in HSE fractionation in the mantle. *Geochimica et Cosmochimica Acta* **71**, 3082–3097 (2007).
154. Backnaes, L. & Deubener, J. Experimental studies on Sulfur solubility in silicate melts at near-atmospheric pressure. *Reviews in Mineralogy & Geochemistry* **73**, 143-165 (2011).
155. Mavrogenes, J. A. & O'Neill, H. S. C. The relative effects of pressure, temperature and oxygen fugacity on the solubility of sulfide in mafic magmas. *Geochimica et Cosmochimica Acta* **63**, 1173-1180 (1999).
156. Keppler, H. Experimental evidence for the source of excess Sulfur in explosive volcanic eruptions, *Science* **284**, 1652-1654 (1999).
157. Van der Wal, D. & Bodinier, J. L. Origin of the recrystallization front in the Ronda peridotite by km-scale pervasive porous melt flow. *Contributions to Mineralogy and Petrology* **122**, 387-405 (1996).
158. Lenoir, X., Garrido, C. J., Bodinier, J. L., Dautria, J. M. & Gervilla, F. The recrystallization front of the Ronda peridotite: Evidence for melting and thermal erosion of subcontinental lithospheric mantle beneath the Alboran basin. *Journal of Petrology* **42**, 141-158 (2001).
159. Bodinier, J. L., Garrido, C. J., Chanefo, I., Bruguier, O. & Gervilla, F. Origin of pyroxenite-peridotite veined mantle by refertilization reactions: Evidence from the Ronda peridotite (Southern Spain). *Journal of Petrology* **49**, 999-1025 (2008).
160. Thordarson, T. & Self, S. Atmospheric and environmental effects of the 1783-1784 Laki eruption: a review and reassessment. *Journal of Geophysical Research* **108** (D1), 4011, doi: 10.1029/2001JD002042 (2003).
161. Bluth, G.J.S., Schnetzler, C.C., Krueger, A.J. & Walter, L.S. The Contribution of explosive volcanism to global sulphur dioxide concentrations. *Nature* **366**, 327-329 (1993)
162. Sobolev, S. V. *et al.* Linking mantle plumes, large igneous provinces and environmental catastrophes. *Nature* **477**, 312-316 (2011).
163. Pollack, J. B. *et al.* Volcanic explosions and climatic change - Theoritical assessement. *Journal of Geophysical Research-Oceans and Atmospheres* **81**, 1071-1083 (1976).
164. Robock, A. Volcanic eruptions and climate. *Reviews of Geophysics* **38**, 191-219 (2000).
165. Self, S., Thordarson, T. & Widdowson, M. Gas fluxes from flood basalt eruptions. *Elements* **1**, 283-287 (2005).
166. Timmreck, C. *et al.* Aerosol size confines climate response to volcanic super-eruptions. *Geophysical Research Letters* **37**, L24705 (2010).
167. Hartmann, G. & Wedepohl, K.H. The composition of peridotites tectonites from the Ivrea complex, northern Italy – Residues from melt extraction. *Geochimica et Cosmochimica Acta* **57**, 1761-1782 (1993).
168. Burnham, O. M., Rogers, N. W., Pearson, D. G., van Calsteren, P. W. & Hawkesworth, C. J. The petrogenesis of the eastern Pyrenean peridotites: an integrated study of their whole-rock geochemistry and Re-Os isotope composition. Geochimica et Cosmochimica Acta **62**, 2293–2310 (1998).
169. Lorand, J.P. & Alard, O. Platinum-group element abundances in the upper mantle: New constraints from in situ and whole-rock analyses of Massif Central xenoliths (France). *Geochimica et Cosmochimica Acta* **51**, 2789-2806 (2001).
170. Lorand, J.P., Alard, O., Luguet, A. & Keays, R.R. Sulfur and selenium systematics of the subcontinental lithospheric mantle: Inferences from the Massif Central xenolith suite (France). *Geochimica et Cosmochimica Acta* **67,** 4137-4151 (2003).
171. Alard, O. *et al.* Volatile-rich Metasomatism in Montferrier Xenoliths (Southern France): Implications for the Abundances of Chalcophile and Highly Siderophile Elements in the Subcontinental Mantle. *Journal of Petrology* **52**, 2009-2045 (2011)
172. Bodinier, J.L., Guiraud, M., Fabriés, J., Dostal, J. & Dupuy, C. Petrogenesis of layered pyroxenites from the Lherz, Freychinéde and Prades ultramafic bodies (Ariége, French Pyrénées) *Geochimica et Cosmochimica Acta* **51**, 279–290 (1987).
173. Sen, I.S. Bizimis, M., Sen, G. & Huang, S. A radiogenic Os component in the oceanic lithosphere? Constraints from Hawaiian pyroxenite xenoliths *Geochimica et Cosmochimica Acta* **75**, 4899–4916 (2011).
174. Müntener, O. The Malenco peridotites (Alps): petrology and geochemistry of subcontinental mantle and Jurassic exhumation during rifting. *PhD thesis*, ETH-Zürich (1997).
